# Testing allele homogeneity: the problem of nested hypotheses

**DOI:** 10.1186/1471-2156-13-103

**Published:** 2012-11-23

**Authors:** Rafael Izbicki, Victor Fossaluza, Ana Gabriela Hounie, Eduardo Yoshio Nakano, Carlos Alberto de Bragança Pereira

**Affiliations:** 1 Department of Statistics, Carnegie Mellon University, Pittsburgh, USA; 2 Department of Statistics, Universidade Federal de São Carlos, São Carlos, Brazil; 3 Department of Psychiatry, University of São Paulo, São Paulo, Brazil; 4 Department of Statistics, University of Braslia, Brasília, Brazil; 5 Department of Statistics, University of São Paulo, São Paulo, Brazil

**Keywords:** Allelic homogeneity test, Bayesian methods, Chi-squared test, Hardy-Weinberg equilibrium, FBST, Monotonicity

## Abstract

**Background:**

The evaluation of associations between genotypes and diseases in a case-control framework plays an important role in genetic epidemiology. This paper focuses on the evaluation of the homogeneity of both genotypic and allelic frequencies. The traditional test that is used to check allelic homogeneity is known to be valid only under Hardy-Weinberg equilibrium, a property that may not hold in practice.

**Results:**

We first describe the flaws of the traditional (chi-squared) tests for both allelic and genotypic homogeneity. Besides the known problem of the allelic procedure, we show that whenever these tests are used, an incoherence may arise: sometimes the genotypic homogeneity hypothesis is not rejected, but the allelic hypothesis is. As we argue, this is logically impossible. Some methods that were recently proposed implicitly rely on the idea that this does not happen. In an attempt to correct this incoherence, we describe an alternative frequentist approach that is appropriate even when Hardy-Weinberg equilibrium does not hold. It is then shown that the problem remains and is intrinsic of frequentist procedures. Finally, we introduce the *Full Bayesian Significance Test* to test both hypotheses and prove that the incoherence cannot happen with these new tests. To illustrate this, all five tests are applied to real and simulated datasets. Using the celebrated power analysis, we show that the Bayesian method is comparable to the frequentist one and has the advantage of being coherent.

**Conclusions:**

Contrary to more traditional approaches, the *Full Bayesian Significance Test* for association studies provides a simple, coherent and powerful tool for detecting associations.

## Background

One of the main goals in genetic epidemiology is the evaluation of associations between specific genotypes or alleles and a certain disease. Association studies are usually performed in a case-control framework in which one or several polymorphisms of candidate genes are evaluated in a group of cases (that is, patients that have a disease) and in a group of controls from the same population (that is, healthy individuals) [1]. The frequencies of each of the genotypes are then computed so that statistical tests that aim at checking for associations between genes and the disease can be performed. The population studied usually must be homogeneous regarding ethnicity, gender distribution and other factors that may bias the results rendering false-positive associations. See [2] for nontechnical summary of reasons that may render false discoveries in case-control studies and [3] for a theoretical analysis of the consequences of population stratification. For more on case-control studies, the reader is referred to [4].

Several statistical tests are usually employed for this scenario. Among them, Cochran-Armitage test for trends [5], homogeneity chi-square tests for contingency tables of both genotypic and allelic frequencies [6], likelihood ratio tests and Wald tests [7] are performed. See, for example, [8] and [9] for a summary of these tests. Some of these statistics are specifically designed to work under assumptions such as dominance models, recessive models or Hardy-Weinberg Equilibrium (HWE). However, a big importance is being given on new methods that are robust to model misspecification, mainly because power is usually small when the model is wrong and type 1 error rates are usually incorrect (see e.g. [7,10,11]).

HWE plays an important role in genetic studies, in particular when testing for allelic homogeneity [12]. The main reason is that the traditional test for allelic homogeneity fails when HWE does not hold, a point to which we will get back later. In words, HWE is a constrain on the genotypic proportions that implies, under some assumptions, stability of the different genotypes over the generations of the population (see e.g. [13] and [14]). These assumptions include, for example, random mating between individuals. For many diseases, random mating is not expected to be satisfied. The same holds for other conditions required for HWE, that in practice may be unrealistic in some situations. In fact, as stated by [15], “a population will never be exactly in HWE”. Hence, the need to design tests that are robust to departures from HWE is evident. A common practice in such problems is to first test HWE, discarding genes that are not in equilibrium. This is done in an attempt of identifying genotyping errors. Such an approach should be avoided, as discussed by [12]. The main reason is that the 2 steps procedure alters type-1 errors. They also emphasize that the correct way to deal with this problem is to inherently account for deviations from HWE with adjusted tests, the approach we take here.

In the present paper, we focus on two hypotheses: 1. homogeneity of the genotypic frequencies; and 2. homogeneity of the allelic frequencies. Usually, data in such studies are summarized in two different ways [9]. The first one consists of a table with the genotypic frequencies of case and control groups. The second, a table with the allelic frequencies. Tables 1 and 2 illustrate this representation using data presented in [16], which was also considered by [12]. Their study was designed to test the hypothesis that GABA_*A*_ sub genes would contribute to a disorder due to methamphetamine use. It is worth noting that Table 2 contains twice as many observations as Table 1. [9] discusses in details the problem of doubling the sample size. In particular, it is shown that methods that “treat alleles as individual entities” [9] have wrong nominal type-1 errors when HWE does not hold. We must recall that the power of a test can increase considerably by increasing the sample size, a nominal increase that can be misleading when it is not reasonable to “treat alleles as individual entities”. This issue we will be discussed in further details later.

**Table 1:** Genotypic frequencies

**Group**	**AA**	**AB**	**BB**	**Total**
Case	55	83	50	188
Control	24	42	39	105

Genotypic frequencies for the data set presented in [16].

**Table 2:** Allelic frequencies

**Group**	**A**	**B**	**Total**
Case	193	183	376
Control	90	120	210

Allelic frequencies derived from Table 1.

The aims of this paper are four-fold: 1 - to describe how the analysis of such data is usually conducted and to emphasize its known flaw (namely lack of robustness to departures from HWE); 2 - to describe one exact frequentist approach which is correct from a classical point of view; 3 - to present a Bayesian method to deal with the problem, and 4 - to advocate the use of the Bayesian solution by demonstrating why this is the best solution compared to the others. The main argument is based on an undesirable logical inconsistency that can happen whenever *p-values* are used to test nested hypotheses. We prove that this does not happen when using the Bayesian method proposed. We also show that the Bayesian and the correct frequentist solutions have comparable power. Simulations and analyses of real data are shown in order to illustrate the problem.

The paper is organized as follows. Section Methods contains three subsections: Usual Procedures, which introduces the notation that is used throughout the paper, discusses the usual methods to deal with the problem and argues why the test for allelic homogeneity is wrong when there are departures from HWE; *A Different Frequentist Test*, which introduces a frequentist test that works even when departures of HWE happen and *Bayesian Solution*, which introduces the FBST approach to solve the problem. Section Results and Discussion first focuses on the issue of the logical incoherence that happens when using the frequentist procedures discussed in the paper and also shows that the same does not happen to the Bayesian method FBST. A brief discussion on Bayes factors is also provided. Finally, we address the question of whether the Bayesian method has good frequentist properties. Section Conclusions summarizes the findings of the paper.

## Methods

Here, we formally describe three different approaches to deal with the problem described: the usual procedure, a correct frequentist proposal and a Bayesian solution.

### Usual Procedures

We begin by describing the statistical model that is used to deal with the problem approached in this paper (namely, product of multinomials) and also how the hypotheses of interest are usually tested in genetic literature. For more details, see [9].

Let $\left(G=\{\text{AA},\text{AB},\text{BB}\}\right)$ be the set of all possible genotypes for the locus of interest. As in Table 3, denote by ***X ***= (*X*_*AA*_,*X*_*AB*_,*X*_*BB*_) and ***Y ***= (*Y*_*AA*_,*Y*_*AB*_,*Y*_*BB*_) the random vectors with the genotypic frequencies from the case and control groups sample, with $\underset{i\in G}{\sum }X_{i}=n$ and $\underset{i\in G}{\sum }Y_{i}=m$ being the total of individuals observed in each group. Also, let ***γ ***= (*γ*_*AA*_,*γ*_*AB*_,*γ*_*BB*_), where *γ*_*i *_is the probability that an individual from the case group has genotype *i*, and ***Π ***= (*Π*_*AA*_,*Π*_*AB*_,*Π*_*BB*_), where *Π*_*i *_is the probability that an individual from the control group has genotype *i*, i∈G. The parametric space is

(1) $$\begin{array}{l} \Theta =\left\{(\gamma_{\text{AA}},\gamma_{\text{AB}},\gamma_{\text{BB}},\Pi_{\text{AA}},\Pi_{\text{AB}},\Pi_{\text{BB}})\in {ℜ}_{+}^{6}:\underset{i\in G}{\sum }\gamma_{i}\right)\\ \left(\;,=\underset{i\in G}{\sum }\Pi_{i}=1\right\}. \end{array}$$

**Table 3:** Population genotypic frequencies

**Group**	**AA**	**AB**	**BB**	**Total**
Case	*x*_*AA*_(*γ*_*AA*_)	*x*_*AB*_(*γ*_*AB*_)	*x*_*BB*_(*γ*_*BB*_)	*n*
Control	*y*_*AA*_(*Π*_*AA*_)	*y*_*AB*_(*Π*_*AB*_)	*y*_*BB*_(*Π*_*BB*_)	*m*

Genotypic frequencies (probabilities).

Considering observations from different individuals to be statistically independent, we have ***X***|*θ *∼* Multinomial*(*n*,***γ***) and ***Y***|*θ *∼ *Multinomial*(*m*,***Π***) with it X and it Y being conditionally independent as well. The likelihood function is then given by

(2) $$L(\theta ;x,y)\propto \underset{i\in G}{\prod }\gamma_{i}^{x_{i}}\underset{i\in G}{\prod }\Pi_{i}^{y_{i}},\theta \in \Theta ,$$

which is the product of two multinomial distributions.

The first hypothesis to be tested (null hypothesis), namely that there is no difference in genotypic frequencies between the groups, may be formally expressed as

(3) $$H_{0}^{G}:\gamma =\Pi .$$

The usual procedure to test $H_{0}^{G}$ is the chi-square test, i.e., the test based on the statistic

(4) $$Q^{G}=\underset{i\in \{\text{AA},\text{AB},\text{BB}\}}{\sum }\left\{\frac{{\left(X_{i}-{\hat{X}}_{i}^{G}\right)}^{2}}{{\hat{X}}_{i}^{G}}+\frac{{\left(Y_{i}-{Ŷ}_{i}^{G}\right)}^{2}}{{Ŷ}_{i}^{G}}\right\},$$

(5) $${\hat{X}}_{i}^{G}=n{\hat{\theta }}_{i}^{G},\;{Ŷ}_{i}^{G}=m{\hat{\theta }}_{i}^{G},$$

where ${\hat{\theta }}_{i}^{G}$ is the maximum likelihood estimator for the genotypic frequency *i* under the hypothesis $H_{0}^{G}$. Under $H_{0}^{G}$, *Q*^*G*^ has asymptotic distribution $\chi_{2}^{2}$ (chi-square distribution with 2 degrees of freedom). Using this fact, it is possible to calculate an asymptotic *p-value*. If one prefers exact tests, Monte Carlo methods can also be used. To sum up, in order to test the first hypothesis, one usually performs a traditional chi-square test of homogeneity to Table 3.

The second hypothesis states that there is no difference in allelic frequencies between the groups. This hypothesis - which will be made formal in the next section - is usually tested by considering the allelic frequencies in both samples, *X*_*A *_= 2*X*_*AA*_ + *X*_*AB*_ and *Y*_*A *_= 2*Y*_*AA*_ + *Y*_*AB*_, as in Table 4 and applying the chi-square test of homogeneity to that table, which has twice as many observations as Table 4.

**Table 4:** Population allelic frequencies

**Group**	**A**	**B**	**Total**
Case	*x*_*A *_= 2*x*_*AA*_ + *x*_*AB*_	*x*_*B *_= 2*x*_*BB*_ + *x*_*AB*_	2*n*
Control	*y*_*A *_= 2*y*_*AA*_ + *y*_*AB*_	*y*_*B *_= 2*y*_*BB*_ + *y*_*AB*_	2*m*

Allelic frequencies from Table 3.

More formally, the statistic considered is

(6) $$Q^{A}=\underset{i\in \{A,B\}}{\sum }\left\{\frac{{\left(X_{i}-{\hat{X}}_{i}^{A}\right)}^{2}}{{\hat{X}}_{i}^{A}}+\frac{{\left(Y_{i}-{Ŷ}_{i}^{A}\right)}^{2}}{{Ŷ}_{i}^{A}}\right\},$$

(7) $${\hat{X}}_{i}^{A}=2n{\hat{\lambda }}_{i}^{A},\;{Ŷ}_{i}^{A}=2m{\hat{\lambda }}_{i}^{A},$$

where ${\hat{\lambda }}_{i}^{A}$ is the maximum likelihood estimator for the allelic frequency *i* under the hypothesis that allelic frequencies are the same in both groups. This statistic is then compared to a $\chi_{1}^{2}$ distribution, or sampled using a Monte Carlo method to calculate the *p-value*. However, in this scenario, the distribution of the test statistic under the null hypothesis is not chi-square unless alleles are statistically independent. In other words, the distribution is chi-square only if a product multinomial model can be applied to Table 4. Essentially, this independence corresponds to the HWE. In fact, [9] formally proves that this is a valid test if, and only if, both groups, case and control, are under HWE. Otherwise, this test is biased: nominal level of significance is different from the real one [17]. [17] also shows how deviations from HWE alter type-I error rates, a point that will also be illustrated in Section Results and Discussion. Therefore, this test should not be used. It is important to note that despite being wrong, it is still widely used in genetic literature nowadays (see e.g. [18], that also discusses some aspects of the lack of robustness of this test). This leads to a larger number of false conclusions than the nominal errors of the procedures.

Applying the traditional tests to data from Table 1, one gets a *p-value* of 0.152 for genotypic association and of 0.049 for allelic association. This means that the evidence we have that the two groups are in genotypic homogeneity is *larger* than the evidence we have that they are in allelic homogeneity. However, if genotypic proportions are the same, allelic proportions must also be the same. This implication will be made formal in Section Results and Discussion. In practice, the first *p-value* being larger than the second implies that one can accept the hypothesis of genotypic homogeneity while rejecting allelic homogeneity, which is a contradiction. For instance, this is the case when the level of significance is 10%, as 0.152 > 0.1 but 0.049 < 0.1. To summarize: we are testing two *nested* hypotheses, that is, the nature of the problem is such that the first hypothesis implies the second. However, even though we reject the second, we do not reject the first. Does the contradiction happen because the allelic test is wrong? Next Section answers this by presenting an exact test for allelic homogeneity that is valid even if HWE does not hold.

### A Different Frequentist Test

Some attempts to correct the above-mentioned allelic test so that it works even when HWE assumption is not met are considered by [8,12,17,19]. See [18] for a summary of these. Also, see [20], that proofs that the test proposed by [17] to correct for departures of HWE is equivalent to the one proposed by [9], which is the Armitage’s trend test [5].

Here we show another solution that has the advantages of being exact, unconditional, and that it can also be calculated in a computationally efficient way, even for large data sets. Moreover, it is defined in the same parametric space Θ as the genotypic test. Essentially, this test is derived by noticing that the hypothesis that allele frequencies are the same in both groups can be written in terms of the original parametric space as

(8) $$H_{0}^{A}:\gamma_{\text{AA}}+\frac{1}{2}\gamma_{\text{AB}}=\Pi_{\text{AA}}+\frac{1}{2}\Pi_{\text{AB}}.$$

Note that this formulation is always true independent of the Hardy-Weinberg equilibrium restriction and does not involve changing neither the sample space nor the parametric space.

The chi-square statistic may be used to test this hypothesis:

(9) $$Q^{A*}=\underset{i\in \{\text{AA},\text{AB},\text{BB}\}}{\sum }\left\{\frac{{\left(X_{i}-{\hat{X}}_{i}^{A*}\right)}^{2}}{{\hat{X}}_{i}^{A*}}+\frac{{\left(Y_{i}-{Ŷ}_{i}^{A*}\right)}^{2}}{{Ŷ}_{i}^{A*}}\right\},$$

(10) $${\hat{X}}_{i}^{A*}=n{\hat{\gamma }}_{i}^{A*},\;{Ŷ}_{i}^{A*}=m{\hat{\Pi }}_{i}^{A*}.$$

Here, ${\hat{\gamma }}_{i}^{A*}$ and ${\hat{\Pi }}_{i}^{A*}$ are the maximum likelihood estimators of genotypic frequencies under $H_{0}^{A}$. They can be found by maximizing

(11) $$\;\begin{array}{l} \;L(\theta ;x,y)\propto \\ {\left(\Pi_{\text{AA}}+\frac{\Pi_{\text{AB}}}{2}-\frac{\gamma_{\text{AB}}}{2}\right)}^{x_{\text{AA}}}\gamma_{\text{AB}}^{x_{\text{AB}}}{\left(1-\Pi_{\text{AA}}-\frac{\Pi_{\text{AB}}}{2}-\frac{\gamma_{\text{AB}}}{2}\right)}^{x_{\text{BB}}}\\ \;\times \Pi_{\text{AA}}^{y_{\text{AA}}}\Pi_{\text{AB}}^{y_{\text{AB}}}{\left(1-\Pi_{\text{AA}}-\Pi_{\text{AB}}\right)}^{y_{\text{BB}}} \end{array}$$

and then using the relations

(12) $${\hat{\gamma }}_{A}^{A*}={\hat{\gamma }}_{\text{AA}}+\frac{1}{2}{\hat{\gamma }}_{\text{AB}};\;{\hat{\gamma }}_{B}^{A*}={\hat{\gamma }}_{\text{BB}}+\frac{1}{2}{\hat{\gamma }}_{\text{AB}};$$

(13) $${\hat{\Pi }}_{A}^{A*}={\hat{\Pi }}_{\text{AA}}+\frac{1}{2}{\hat{\Pi }}_{\text{AB}};\;{\hat{\Pi }}_{B}^{A*}={\hat{\Pi }}_{\text{BB}}+\frac{1}{2}{\hat{\Pi }}_{\text{AB}}.$$

Maximization of Equation (3) can be efficiently done by using numerical methods such as Newton’s method [21], which are already implemented in most statistical and mathematical softwares such as R and MATLAB. To calculate *p-values*, the statistic *Q*^*A*∗^ can then be compared to a $\chi_{1}^{2}$ distribution or, if one wishes to perform an exact test (the approach we take here), sampled using Monte Carlo methods. That is, one can generate several values of *Q*^*A*∗^under the null hypothesis and compute the proportion of these that are larger than the observed statistic on the sample. This is the (estimate of the) exact *p-value*. Confidence intervals can be obtained for it by using a normal approximation to the binomial distribution. Note that the dimension of the parametric space is 4 and under the null hypothesis it becomes 3. Hence, the number of degrees of freedom of the distribution of the chi-square statistic is $\text{dim}(\Theta )-\text{dim}(H_{0}^{A})=4-3=1$. This is also the number of degrees of freedom of the chi-square for the allelic test described before.

This test is very similar to the ones recently introduced by [7], except that the statistics used are different (Wald statistic, score statistic and maximum profile likelihood ratio), and results are asymptotic: chi squared approximation is used. Even though these tests are asymptotically equivalent, in order to illustrate our points it is important to have exact tests here.

The allelic *p-value* for data from Table 3 is 0.069. It is surprising that despite the fact this test is correct, this *p-value* is still smaller than 0.152 - the *p-value* for genotypic association. Hence, incoherence remains even when correcting the traditional allelic test. We note that the *p-value* found by [12] for this same data set using corrected allelic test is 0.066, which also does not remove the contradiction. Note that here we use the exact test, hence this is not a problem of using an approximation. In the Section Results and Discussion we present other data sets in which this incoherence happens, showing that this problem is not unique to the particular data we chose to illustrate the point. Next Section is devoted to present a framework where this kind of contradiction does not happen.

### Bayesian Solution

Bayesian methods are the alternative inductive way to deal with such a problem. These methods are widely used nowadays because they allow prior knowledge from the researcher and scientific community to be incorporated into the analysis (see [22] for applied examples of these methods in genetics) and, contrary to usual classical procedures, they do not require large samples for the analysis to be correct. That is, optimality of the procedure does not rely on asymptotic considerations. Many Bayesian methods designed to deal with precise hypotheses, i.e., hypotheses which have lower dimension than the parametric space, have been developed. Precise hypotheses must have a different treatment in Bayesian statistics: in general, they have zero posterior probability, so that they would always be rejected when using traditional methods. One way to deal with this problem is to assign a positive prior probability to null hypothesis [23], but this may seem a rather *ad hoc* solution and may lead to some inconsistencies [24]. Another approach is to use Bayes factors [25], a point to which we will get back later in the paper (see Section Results and Discussion).

In this paper, we choose to use the FBST (*Full Bayesian Significance Test*), a procedure introduced by [26]. This method was also used by [27]. The test is based on the *e-value* statistic, a Bayesian measure of evidence designed to evaluate sharp null hypotheses. In order to apply this method, we begin by specifying a prior density in the complete parametric space Θ, *f*(***θ***). We note that it is not necessary to attribute different probability to each of the hypothesis: it is only necessary to specify *f*(***θ***). This is not the case for Bayes factors, where specification of different probability distribution inside each of the hypothesis of interest is needed. After observing data ***x***, let *f*(***θ***|***x***) be the posterior density of the parameter *θ*. The posterior density is given by

(14) f(θ|x)∝f(θ)L(θ;x).

Suppose one is interested in testing the null hypothesis *H*: ***θ ***∈* Θ*_0_. Define the Tangential Set to the null hypothesis as

(15) $$T_{x}^{H}=\{\theta \in \Theta :f(\theta |x)>\underset{\Theta_{0}}{\text{sup}}f(\theta |x)\}.$$

The measure of evidence proposed, the ite-value, is defined by

(16) $$ev_{x}(H)=1-P(\theta \in T_{x}^{H}|x).$$

In words, *e-value* is the posterior probability of the subset of the parametric space consisting of points with lower posterior density than the maximum achieved under *H*. It is interesting to note the duality between *p-values* and *e-values*: while the former are tails in the sample distribution from the observed values under the null hypothesis, the latter are tail areas in the posterior distribution from the sharp hypothesis. *E-values* are easy to be calculated and successful papers that use FBST procedure in genetics include [14,28,29]. For more on *e-values*, the intuition behind it, asymptotic consistency results, and decision-theoretic considerations see [30]. High *e-values* indicate high evidence in favor of the hypothesis, while low *e-values* indicate that the hypothesis is false.

Implementation of the FBST procedure requires two simple steps, which can be performed numerically: 

• *Optimization* - Finding the supremum of the posterior distribution under the null hypothesis, ${\text{sup}}_{\Theta_{0}}f(\theta |x)$. This is usually done by using built-in functions from statistical packages such as R.

• *Integration* - Integrating the posterior density over the Tangential Set, $T_{x}^{H}$. This step can be done by sampling from the posterior distribution by using methods such as MCMC. For the problem considered here, a usual Monte Carlo method is enough to efficiently sample from the posterior.

More details on the implementation of the FBST procedure can be found in [26]. To perform the complete FBST procedure one also needs to set a cut-off point, that is, one must say what a “small” *e-value* means. Several approaches are available: 

• Empirical power analysis [31]

• Reference sensitivity analysis and paraconsistent logic [32].

• [30] relate *e-values* to *p-values*.

• Bayesian decision-theoretic approach [33], by the specification of a loss function that gives origin to FBST procedure.

• An asymptotically consistent threshold for a given confidence level ([34] and [31]).

The prior distribution for ***γ ***in the routine that was implemented and is available in the website is a Dirichlet distribution, as well as the prior distribution for ***Π***. The family of the Dirichlet priors is widely used in this scenario once it is both broad enough to contemplate a huge number of different possible prior information and yet very easy to be dealt with both mathematically and computationally. Here, the two priors are considered to be independent, and in the implementation we provide online (see Conclusions) the (hyper)parameters (*a*_*AA*_,*a*_*AB*_,*a*_*BB*_,*b*_*AA*_,*b*_*AB*_,*b*_*BB*_) are set by the user. That is,

(17) $$f(\theta )\propto \underset{i\in G}{\prod }\gamma_{i}^{a_{i}-1}\underset{i\in G}{\prod }\Pi_{i}^{b_{i}-1},\theta \in \mathrm{\Theta .}$$

Note that in this case the posterior distribution is also the product of two independent Dirichlet distributions (once they are conjugate with the multinomial distribution). Their parameters are (*x*_*AA*_ + *a*_*AA*_,*x*_*AB*_ + *a*_*AB*_,*x*_*BB*_ + *a*_*BB*_) and (*y*_*AA*_ + *b*_*AA*_,*y*_*AB*_ + *b*_*AB*_,*y*_*BB*_ + *b*_*BB*_) respectively. Simulation of the Dirchlet distribution can be efficiently done by sampling from Gamma distributions; see [35] for details. Note that the case where all (hyper)parameters (*a*_*i*_ and *b*_*i*_) are equal to 1, *θ* is uniformly distributed *a priori*.

**Figure 1:**
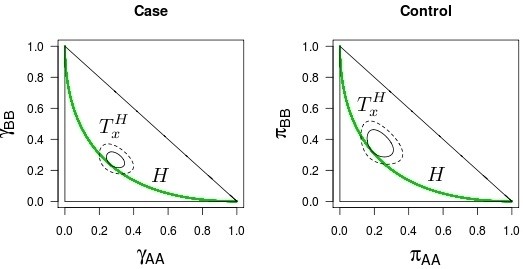
***Full Bayesian Significance Test *for HWE: real data.** Geometric representation of the HWE hypothesis (green curve), FBST tangential set (continuous ellipsis) and 99% credible set (dashed ellipsis): data from real samples.

**Figure 2:**
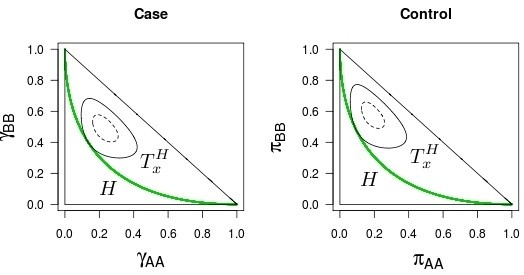
***Full Bayesian Significance Test *for HWE: simulated data.** Geometric representation of the HWE hypothesis (green curve), FBST tangential set (continuous ellipsis) and 99% credible set (dashed ellipsis): data from simulated samples (case 26 from Table 6).

The FBST procedure can be used in general, not only for testing allelic homogeneity. In particular, it can be used to test Hardy-Weinberg equilibrium, as shown by [26] and [14]. In order to illustrate the procedure, Figure 1 shows the Hardy-Weinberg hypothesis line and the Tangential Sets for both case and control groups from the data set presented by [16]. Figure 2 does the same for simulated data (not under Hardy-Weinberg equilibrium). They also show the 99% HPD (Highest Posterior Density) sets. For the sake of neutrality, the prior distribution we use is the product of two independent Dirichlet distributions with parameters (1,1,1), i.e., the uniform distribution in Θ. Hence, the posterior distribution is proportional to the likelihood function, that is,

(18) $$f(\theta |x,y)\propto \underset{i\in G}{\prod }\gamma_{i}^{x_{i}}\underset{i\in G}{\prod }\Pi_{i}^{y_{i}},\theta \in \mathrm{\Theta .}$$

We see that while both groups from Figure 1 seem to be under HWE (in this case, tangential sets have small probabilities, and therefore *e-values* are large), the ones from Figure 2 seem to be far from the equilibrium (in this case, tangential sets have large probabilities, and therefore *e-values* are small).

When testing genotypic and allelic homogeneity using FBST and uniform priors (*a*_*i *_=* b*_*i *_= 1 for all *i*’s in the Dirichlet distribution), we obtain *e-values* of 0.434 and 0.493 respectively. Hence, contrary to what happens to *p-values*, there is more evidence in favor of the allelic homogeneity hypothesis than there is in favor of the genotypic homogeneity hypothesis. Therefore the contradiction of not rejecting the first hypothesis while rejecting the second one cannot happen for any cutoff that is chosen. In fact, as we will show in next Section, this is a property of the FBST procedure: the undesirable contradiction can never happen.

## Results and Discussion

We begin this Section by summarizing the results of the analyses for data presented in [16], which were presented during the exposition of the concepts throughout the paper. Results are shown in Table 5. The notation for this table is as follows: *p*^*G*^ is the traditional *p-value* for genotypic association; *e*^*G*^ is the *e-value* for genotypic association; $p_{U}^{A}$ is the usual (wrong) *p-value* for allelic association; *p*^*A *^is the *p-value* for allelic association proposed in this paper; and *e*^*A*^ is the *e-value* for allelic association. This table also includes the results (both *p-values* and *e-values*) for the test of the hypothesis of HWE for case and control group.

**Table 5:** Analysis of real data

**Genotypes**		**Alleles**			**Hardy-Weinberg**			
***p***^***G***^	***e***^***G***^	$p_{U}^{A}$	***p***^***A***^	***e***^***A***^	***p***^***HW***^	***e***^***HW***^	***p***^***HW***^	***e***^***HW***^
0.152	0.434	0.049	0.069	0.493	0.111	0.276	0.060	0.165

Significance indices for homogeneity for data presented in Table 2.

**Table 6:** Analysis of simulated data

	**Genotypes**		**Alleles**			**Hardy-Weinberg**			
						**Case**		**Control**	
	***p***^***G***^	***e***^***G***^	$p_{U}^{A}$	***p***^***A***^	***e***^***A***^	***p***^***HW***^	***e***^***HW***^	***p***^***HW***^	***e***^***HW***^
**Genotypic Homogeneity**									
1	**0.408**	0.773	**0.197**	**0.189**	0.786	0.540	0.832	0.819	0.971
2	0.588	0.897	0.648	0.684	0.997	0.030	0.090	0.001	0.002
3	0.478	0.826	0.483	0.510	0.980	0.496	0.793	0.035	0.119
4	**0.912**	0.996	**0.709**	**0.689**	0.997	0.172	0.377	0.122	0.287
5	**0.836**	0.985	**0.578**	**0.554**	0.985	0.224	0.464	0.170	0.378
6	**0.989**	1.000	**0.926**	**0.903**	1.000	0.000	0.000	0.000	0.000
7	**0.187**	0.494	**0.100**	**0.068**	0.498	0.027	0.081	0.044	0.124
8	**0.652**	0.929	**0.444**	**0.416**	0.953	0.338	0.626	0.104	0.257
9	**0.620**	0.916	**0.510**	**0.494**	0.976	0.192	0.422	0.761	0.955
10	0.565	0.888	0.923	0.912	1.000	0.001	0.003	0.057	0.153
**Allelic Homogeneity**									
11	0.008	0.034	0.325	0.291	0.893	0.494	0.790	0.001	0.003
12	0.000	0.000	0.067	0.057	0.442	0.068	0.190	0.000	0.000
13	0.002	0.013	0.151	0.114	0.629	0.989	1.000	0.000	0.000
14	0.001	0.003	0.923	0.918	1.000	0.174	0.400	0.000	0.000
15	0.113	0.342	0.844	0.833	1.000	0.989	1.000	0.006	0.014
16	0.020	0.086	0.559	0.547	0.985	0.174	0.395	0.015	0.040
17	0.001	0.006	0.147	0.129	0.683	0.129	0.319	0.002	0.005
18	0.040	0.149	0.501	0.462	0.970	0.871	0.986	0.001	0.002
19	0.026	0.106	1.000	1.000	1.000	0.760	0.955	0.000	0.000
20	0.001	0.002	0.446	0.379	0.939	0.733	0.938	0.000	0.000
**No Homogeneity**									
21	0.000	0.000	0.925	0.928	1.000	0.000	0.000	0.015	0.045
22	**0.843**	0.987	**0.646**	**0.618**	0.993	0.055	0.153	0.141	0.333
23	0.062	0.219	0.104	0.124	0.661	0.989	1.000	0.007	0.028
24	**0.669**	0.939	**0.403**	**0.408**	0.955	0.994	1.000	0.621	0.882
25	0.000	0.000	0.000	0.000	0.003	0.000	0.000	0.771	0.958
26	0.105	0.331	0.017	**0.047**	0.403	0.001	0.001	0.001	0.001
27	0.000	0.000	0.000	0.000	0.012	0.072	0.197	0.010	0.033
28	0.180	0.485	0.230	0.233	0.835	0.310	0.598	0.324	0.602
29	**0.134**	0.387	**0.068**	**0.045**	0.389	0.045	0.128	0.063	0.170
30	**0.807**	0.980	**0.522**	**0.517**	0.980	0.806	0.971	0.713	0.933

Results of the simulations under three different scenarios: genotypic homogeneity, allelic (but not genotypic) homogeneity and no homogeneity at all. Bold p values indicate incoherence.

Hence, it is reasonable to expect that *p-values*, as well as any other measure of evidence, should be such that $p(H_{0}^{A})\geq p(H_{0}^{G})$. To sum up, there should be more evidence in favor of $H_{0}^{A}$ than in favor of $H_{0}^{G}$. In fact, this is what motivates the tests proposed by [7]. More generally, if we have two nested hypotheses, *A *⊆* B *⊆ Θ, it would be desirable to have *p*(*B*) ≥* p*(*A*). That is, one should always believe that *B* is at least as plausible as *A*. It is worth noting that this inequality must hold if one wants to guarantee that for any significance level *α* the rejection of *B* will imply the rejection of *A*. In other words, *p*(*B*) should always be greater than *p*(*A*) so that one will never conclude that *A* holds but *B* does not, which is, as we showed, logically impossible.

Even though this logical coherence is desirable, the analysis of data presented by [16] (Table 5) shows that this property is not achieved neither when using the traditional *p-value* for allelic frequencies, nor when using the alternative test presented here. Hence, depending on the level of significance used (for example, 10%), one can conclude that genotypic homogeneity holds, but allelic homogeneity does not. This leads one to a logical contradiction that may be embarrassing for the researcher when showing his results to scientific community. Some authors (e.g. [36-39]) have already noticed that *p-values* cannot be used as a measure of evidence because they do not respect this property. Attempts to correct frequentist tests so that they are coherent have been tried in some specific situations such as Analysis of Variance [40], but no general procedure could be obtained.

On the other hand, *e-values* are monotone in the set of all possible hypotheses. This can be seen by noting that

(19) $$\Theta_{0}\subseteq \Theta_{0}^{\prime }\subseteq \Theta \Rightarrow T_{x}^{\Theta_{0}^{\prime }}\subseteq T_{x}^{\Theta_{0}}\Rightarrow ev_{x}(\Theta_{0})\leq ev_{x}(\Theta_{0}^{\prime }).$$

For the problem considered here, this means that $ev_{x}(H_{0}^{G})\leq ev_{x}(H_{0}^{A})$ will hold for all datasets. Hence, one will always have at least as much evidence in favor of $H_{0}^{A}$ as in favor of $H_{0}^{G}$, and therefore when performing the FBST procedure (that is, comparing the *e-values* with a given cutoff) one will never fall into the logical contradiction of rejecting $H_{0}^{A}$ while not rejecting $H_{0}^{G}$. Equation 4 proves that the incoherence can never happen when using the FBST. Table 5 shows that this inequality indeed holds for the data presented. It is also interesting to note that in the case of nested hypotheses, FBST provides an intrinsic penalty that can be used for model selection [41].

In Table 6, one can find similar results on simulated data. Data was simulated in three different conditions: 1 - under genotypic homogeneity (and, therefore, allelic homogeneity), 2 - under only allelic homogeneity and 3 - under neither allelic nor genotypic homogeneity. Bold *p-values* indicate situations in which there is incoherence in the sense described here. Note that, as it was expected due to the proof that was given, none of the samples have incoherence when using analyses provided by *e-values*.

As mentioned before $H_{0}^{G}$ implies $H_{0}^{A}$ in the sense that *if genotypic frequencies are the same in both groups then allelic frequencies must also be the same*. In other words, it is impossible for the allelic frequencies to be different if the genotypic frequencies are equal. This can be formally seen by noting that

(20) $$\begin{array}{lll} H_{0}^{G}\;\text{true}\Rightarrow \gamma_{i} & =\Pi_{i}\forall i\in G\Rightarrow \gamma_{\text{AA}}+\frac{1}{2}\gamma_{\text{AB}}\; & \;\\ & =\Pi_{\text{AA}}+\frac{1}{2}\Pi_{\text{AB}}\Rightarrow H_{0}^{A}\;\text{true}.\; & \; \end{array}$$

An important question is why we use FBST methodology rather then standard Bayes factors, the traditional Bayesian procedure to test sharp hypotheses [25]. The reason is that, contrary to *e-values*, Bayes factors are also not monotonic when dealing with sharp hypotheses as we will show here. In order to calculate Bayes factors, one must first assign a probability distribution for the parameters under each of the hypothesis of interest. In the problem we deal with, this means it is necessary to assign probabilities for *θ* under Θ, $H_{0}^{G}$ and $H_{0}^{A}$. The Bayes factor for hypothesis *H* is then defined to be $\frac{P(\text{data}|H)}{P(\text{data}|\Theta )}$[38]. For the real dataset presented in [16] (Table 1), when using uniform probabilities for *θ* in Θ, $H_{0}^{G}$ and $H_{0}^{A}$ we have a Bayes factor of 6.63 in favor of $H_{0}^{G}$, while of 0.28 in favor of $H_{0}^{A}$, so that lack of monotonicity remains. The main reason for this is that it is not necessarily true that $P(\text{data}|H_{0}^{G})\leq P(\text{data}|H_{0}^{A})$. See [38] for a different example where this happens. An informal explanation of the lack of monotonicity is given by [38]: *“What the Bayes factor actually measures is the change in odds in favor of the hypothesis when going from the prior to the posterior”*. Note that even though they are not monotonic, Bayes Factors provide a great tool for model selection [42], a point which we further discuss in the conclusions. One may also argue about the merits of using FBST as a genuine Bayesian procedure rather than traditional Bayes factors. We advocate that while Bayes factors are primarily motivated by the epistemological framework of Decision Theory and *p-values* are supported by Popperian falsificationism, *e-values* and FBST are supported by the framework of Cognitive Constructivism. The reader is referred to [43-45] for more epistemological considerations and comparisons of these methods. It is also interesting that FBST can also be justified as a minimization procedure of a loss function, as shown by [33]. This makes *e-value* also compatible with standard Decision Theory and therefore traditional Bayesian statistics. We emphasize that whenever hypotheses are not sharp, posterior probabilities are usually more adequate.

**Figure 3:**
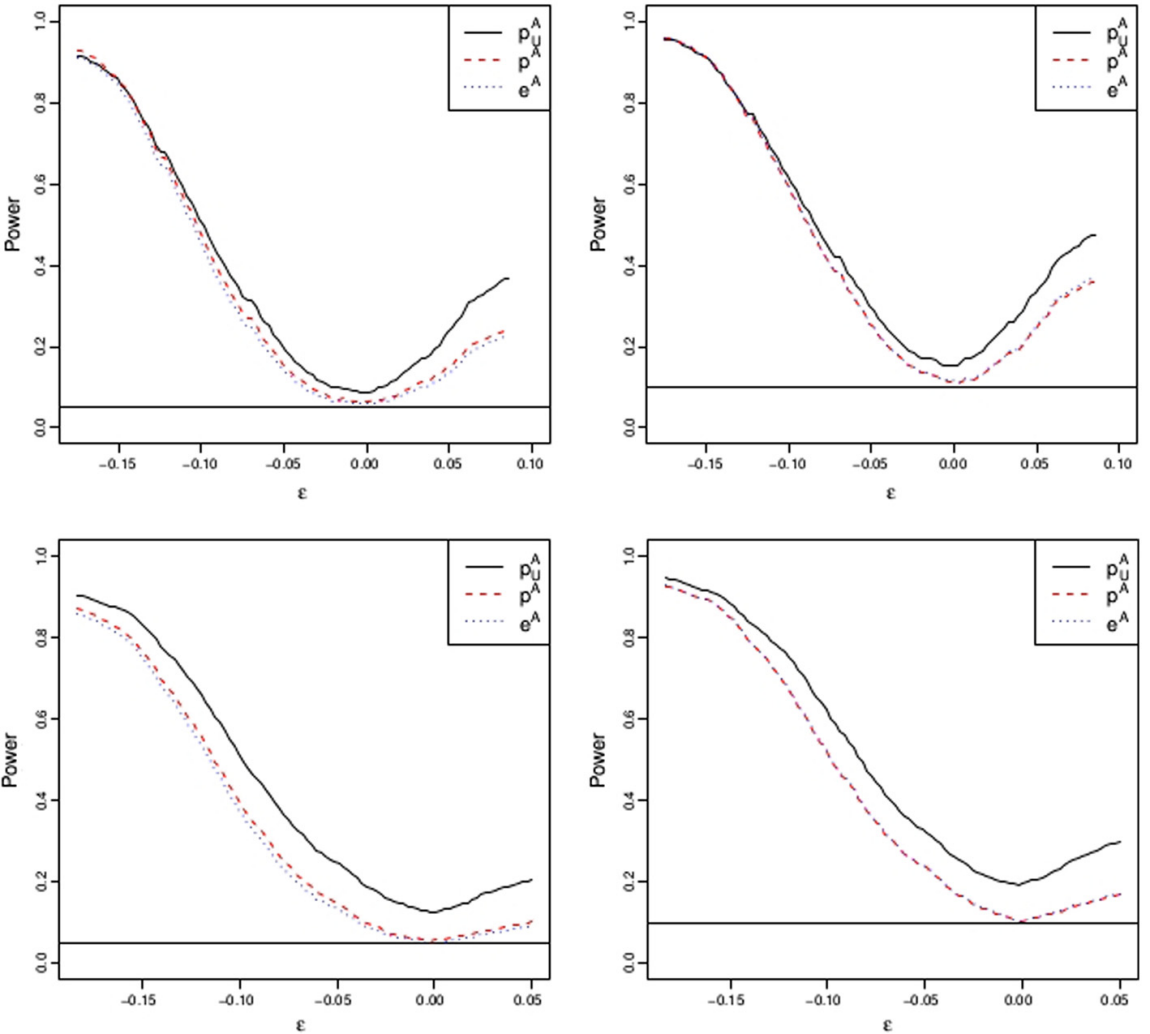
**Power analysis of *Full Bayesian Significance Test*.** Comparison of power of different tests for allelic homogeneity. Horizontal lines show level of significance. Topleft: *γ*_*AA *_= 1/5,*γ*_*AB *_= 2/5,*Π*_*AA *_= 1/4,*α *= 5*%*, topright: *γ*_*AA *_= 1/5,*γ*_*AB *_= 2/5,*Π*_*AA *_= 1/4,*α *= 10*%*, bottomleft: *γ*_*AA *_= 1/3,*γ*_*AB *_= 1/5,*Π*_*AA *_= 1/3,*α *= 5*%*bottomright: [*γ*_*AA *_= 1/3,*γ*_*AB *_= 1/5,*Π*_*AA *_= 1/3,*α *= 10*%*.

We end up this Section by answering the question of whether FBST procedure has good power properties. Even though this is not of primary interested in this work and is not a relevant question for most orthodox Bayesians, we indicate that this Bayesian procedure has good frequency properties. In order to do this, we fix different values for *γ*_*AA*_,*γ*_*AB*_ and *Π*_*AA*_. We then set *Π*_*AB*_ to be 2(*γ*_*AA*_ + 1/2*γ*_*AB*_−*Π*_*AA*_−*ε*) for different values of *ε.ε *quantifies how far from allelic homogeneity the population is. The particular case *ε *= 0 corresponds to allelic homogeneity, that is, to a true hypothesis. For each value of *ε*, we simulate 100 data sets with 100 samples of cases and 100 samples of controls group. We then calculate the proportion of samples in which allelic homogeneity was rejected according to each criteria. We use levels of significance of 5% and 10%. The relationship provided by [30] is used to determine the cutoffs for *e-values* that make FBST have the desired level of significance. Results are shown in Figure 3. These graphs indicate that the usual test for allelic homogeneity has a larger power than the others. This conclusion is misleading, once the size of the test is *not* the nominal one, as we discuss in Section Usual Procedures. This can be seen by looking at the curve at *ε *= 0 and noting that the power (which for *ε *= 0 is the size of the test) is larger than 5% and 10% respectively. For more simulations regarding this test power, the reader is referred to [17]. This figure also shows that the power of the frequentist allelic test proposed here and the FBST test are virtually the same: even though FBST structure guarantees coherence in the results and frequentist tests do not have the property, their power are very close to each other. Hence, the FBST procedure also has good frequency properties.

## Conclusions

Although the traditional approach of doubling the sample size to test allelic homogeneity hypothesis was already shown to be incorrect when Hardy-Weinberg equilibrium is not met, many recent articles in biology still use it. As Figure 3 illustrates by using power analysis functions, the nominal level of significance for the allelic usual test is not attained: at zero in the x-axis, the power is larger than 5%, contrary to the alternative ones. We have shown in this paper that a logical inconsistency that happens when using such procedure remains even when using adjusted frequentist tests. The main point of this inconsistency is the fact that if two vectors are equal any function of them must maintain the equality. The fact that even when using an exact approach incoherence remains hints that the problem is the change of dimension when going for global homogeneity to partial homogeneity: genotypic homogeneity is in dimension 2 (two degrees of freedom) and allelic homogeneity is in dimension 1 (one degree of freedom). As Wald Tests, Likelihood Ratio Tests, and Chi-Square tests are asymptotically equivalent, it is also expected that contradictions may happen to all of them.

Similar incoherences of *p-values* in other situations have already been reported in the literature. As a simple ANOVA-like example, suppose we wish to compare the means of independent random variables from 3 different groups, *μ*_1_,*μ*_2_ and *μ*_3_. If we assume their distribution is normal with variance 1 and the sample means in each group (sufficient statistics) are −0.192,0.015 and 0.017, the likelihood ratio *p-value* for the hypothesis *μ*_1_ =* μ*_2_ is 0.037. On the other hand, when testing *μ*_1_ =* μ*_2_ =* μ*_3_ we get a *p-value* of 0.054. Hence, at the level of 5*%*, the first hypothesis is rejected, but the second one is not. This makes it debatable whether it reasonable to use them as measures of evidence [37]. On the other hand, if we use the improper prior *f*(*μ*_1_,*μ*_2_,*μ*_3_)∝1, the *e-values* are 0.232 and 0.121, respectively. Hence the contradiction cannot happen for any cutoff.

As probabilities are monotonic, traditional Bayesian tests based on posterior probability calculations do enjoy monotonicity property, however using them here may be problematic because the hypotheses of interest are sharp. Mixed continuous-discrete distributions are needed in this case. Bayes Factors, on the other hand, were shown to be not monotonic. This does not invalidate its use: in fact, as pointed out by [38] and [42], Bayes Factors provide a great tool for model selection. One of the reasons for this is that parsimonious models can have better predictive power than complex models [46].

The FBST computation always is performed in the full space that has dimension 4. Hence subhypotheses should coherently follow the orientation of the main hypothesis. Moreover, there is no need of specifying special priors for each of the null hypotheses, only for the whole parametric space Θ. It can also be easily implemented. The problem with the FBST is that the values of the significance index, “e”, are related to the dimension and increase as the dimension increases. However, in [47] it is shown how “e” relates with “p”. This allows one to look for the corresponding e-value for 5% of significance for instance. Another point in favor of the FBST is that its power is almost the same as the best frequentist test. Moreover, it is correct even when HWE does not hold. It is important to remember that e-values are probabilities of subsets of the parameter spaces although p-values are probabilities of sets (tails) of the sample spaces. On the other hand one must understand that hypotheses are statements about points of the parameter space and *not* of the sample space: May this explain the reason why the e-values, contrary to p-values, are coherent in all situations?

Using the R Software, a routine that performs all the tests considered in this paper can be downloaded on http://www.ime.usp.br/∼cpereira/programs/nested.r

## Competing interests

The authors declare that they have no competing interests.

## Authors’ contributions

RI wrote the original manuscript. AGH was responsible for finding real data for the problem, as well as discussing the methods from a biological point view. AGH and RI did the literature survey. EYN, RI and VF worked on the mathematics of the methods as well as implemented them. CAdeBP first noticed the lack of monotonicity of the previous approaches and introduced the *FBST* as a way of solving the problem. All authors revised, read and approved the final manuscript.
